# Bat adaptations in inflammation and cell death regulation contribute to viral tolerance

**DOI:** 10.1128/mbio.03204-23

**Published:** 2025-02-21

**Authors:** Subham Das, Disha Jain, Priyansh Chaudhary, Rita M. Quintela-Tizon, Arinjay Banerjee, Sannula Kesavardhana

**Affiliations:** 1 Department of Biochemistry, Indian Institute of Science, Bengaluru, Karnataka, India; 2 Vaccine and Infectious Disease Organization (VIDO), University of Saskatchewan 7235 , Saskatoon, Saskatchewan, Canada; 3 Department of Veterinary Microbiology, University of Saskatchewan 7235 , Saskatoon, Saskatchewan, Canada; 4 Department of Biology, University of Waterloo, Waterloo, Ontario, Canada; 5 Department of Laboratory Medicine and Pathobiology, University of Toronto, Toronto, Ontario, Canada; 6 Department of Biochemistry and Molecular Biology, University of British Columbia, Vancouver, British Columbia, Canada; Albert Einstein College of Medicine, Bronx, New York, USA

**Keywords:** bats, zoonotic viruses, cell death, viral tolerance, inflammation, pyroptosis, necroptosis, gasdermins

## Abstract

Bats are reservoirs for multiple viruses, some of which are known to cause global disease outbreaks. Virus spillovers from bats have been implicated in zoonotic transmission. Some bat species can tolerate viral infections, such as infections with coronaviruses and paramyxoviruses, better than humans and with less clinical consequences. Bat species are speculated to have evolved alongside these viral pathogens, and adaptations within the bat immune system are considered to be associated with viral tolerance. Inflammation and cell death in response to zoonotic virus infections prime human immunopathology. Unlike humans, bats have evolved adaptations to mitigate virus infection-induced inflammation. Inflammatory cell death pathways such as necroptosis and pyroptosis are associated with immunopathology during virus infections, but their regulation in bats remains understudied. This review focuses on the regulation of inflammation and cell death pathways in bats. We also provide a perspective on the possible contribution of cell death-regulating proteins, such as caspases and gasdermins, in modulating tissue damage and inflammation in bats. Understanding the role of these adaptations in bat immune responses can provide valuable insights for managing future disease outbreaks, addressing human disease severity, and improving pandemic preparedness.

## INTRODUCTION

**Fig 1:**
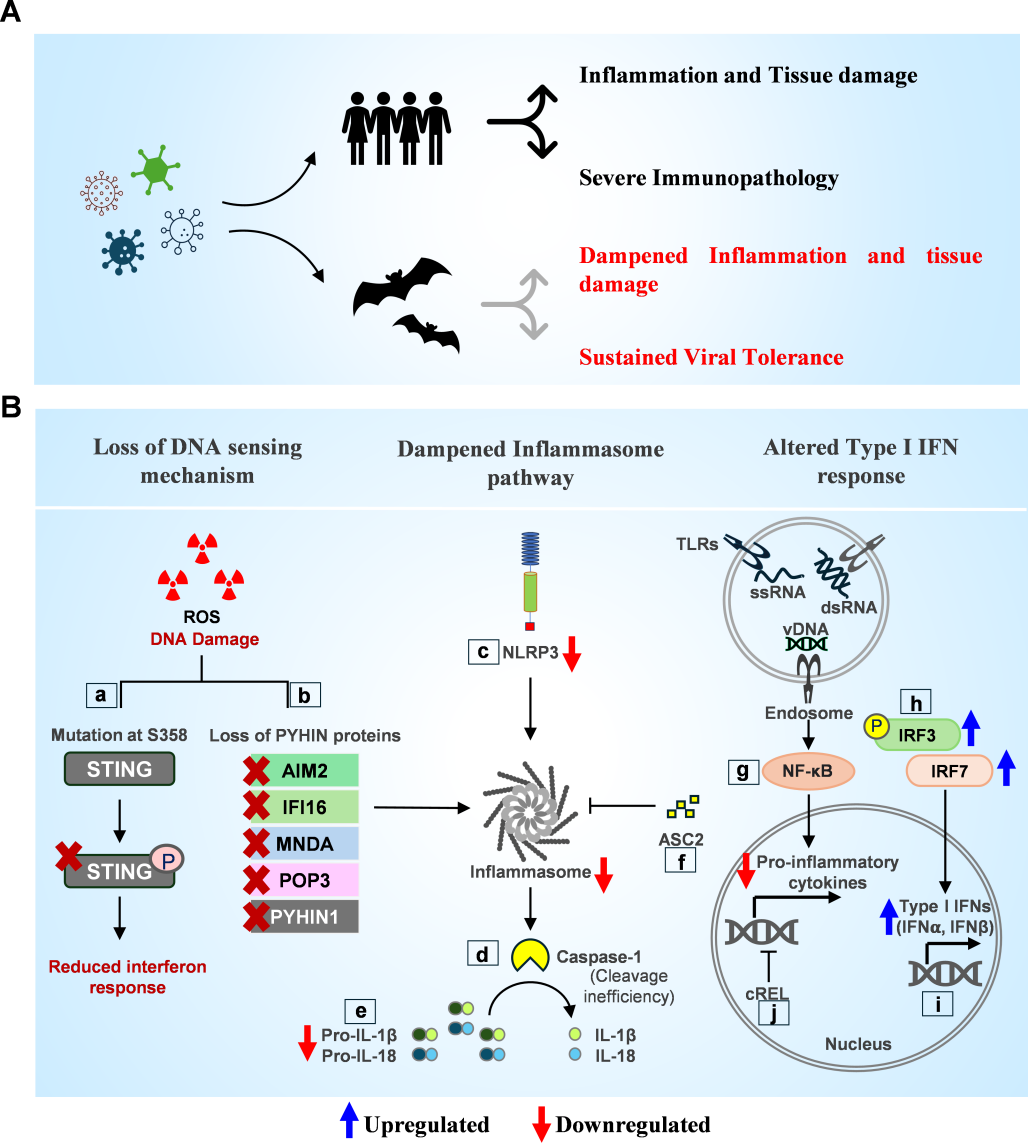
Unique immune adaptations in bats as viral reservoirs. (**A**) The schematic represents bat and human-specific responses to virus infection. Zoonotic virus infections cause cellular and tissue damage and inflammation-driven immunopathology in humans. Unlike humans, bats do not manifest severe disease outcomes after viral infections and exhibit a balanced viral tolerance. (**B**) (a, b) Bats have adapted to regulate the STING (stimulator of interferon genes) pathway to mitigate cGAS-STING-dependent DNA sensing and activation of inflammatory response. *T. brasiliensis* possesses a point mutation at the S358 of the STING, dampening its phosphorylation and reducing the downstream IFN (Interferon) response. Bats have lost complete PYHIN (PYRIN and HIN domain) family genes, leading to a reduced NLRP3 (NOD-like receptor family pyrin domain containing 3)-dependent inflammasome response. (c) NLRP3 inflammasome activation is dampened at transcriptional and protein levels. (d, e) Bat caspase-1 and interleukin-1β (IL-1β) are dysfunctional in *P. alecto*. Bat caspase-1 is positively selected at the 365th residue within the p10 domain, required for the homodimerization of caspase-1 and subsequent cleavage of IL-1β and gasdermin D (GSDMD). (f) *P. alecto* ASC2 (apoptosis-associated speck-like protein containing a CARD) downregulates NLRP3 inflammasome activation during SARS-CoV infection. (g) Bats have altered specificity in TLRs, activating the downstream NF-κB (nuclear factor kappa-light-chain-enhancer of activated B cells) pathway to regulate the expression of inflammatory cytokines (TNF-α, IL-1β, IL-18, and IL-6). (h, i)interferon regulatory factor 3 (IRF3) has undergone positive selection to mount a robust antiviral response through the type I IFN expression. Additionally, the unique expression patterns of IRF7 upregulate the IFN response. (j) c-REL is positively selected in *E. fuscus,* suppressing TNFα and IL-8 expression, which may limit inflammation and reduce the risk of immunopathology during viral infections.

The increased incidence of pandemics and viral outbreaks in recent years has drawn attention to understanding host-virus interactions in recognized reservoir species and developing prevention measures. Zoonotic transmission, where viruses spill over from animal hosts to humans, has been a primary cause of recent human virus outbreaks. Approximately 60% of infectious diseases affecting the human population originate from nonhuman animal sources, including bats, pigs, civet cats, and birds (1, 2). Of note, bats and birds are the primary reservoirs for pathogenic RNA viruses (1–3), including coronaviruses like severe acute respiratory syndrome coronavirus (SARS-CoV), Middle East respiratory syndrome coronavirus (MERS-CoV), SARS-CoV-2, influenza A virus, Hendra virus, Nipah virus, Ebola virus, and Marburg virus (4–11). Despite harboring these viruses, naturally and experimentally infected bats generally exhibit minimal clinical symptoms (Fig. 1A), raising intriguing questions about their immune adaptations that allow them to tolerate diverse viral species without significant immunopathology (12–15). The specific mechanisms conferring viral tolerance in bats and other reservoir hosts remain less studied. The mechanisms underlying bat tolerance to viral infections are likely multifaceted, perhaps involving sophisticated immune adaptations and controlled inflammatory responses (13, 14, 16).

Bats are a diverse order with over 1,480 species belonging to the order Chiroptera (17). Bats were categorized into megabats and microbats, typically based on their body size and echolocation. With advancements in next-generation sequencing, bats have been reclassified into two suborders: Yangochiroptera and Yinpterochiroptera (18). Bats have a unique ability among mammals. Their sustained flight leads to increased metabolic demands and activity. In addition, bats also have extended lifespans compared to other mammals of a similar size (19–21). Perhaps the evolutionary adaptations of bats, including a high metabolic rate and extended lifespan, likely contribute to their ability to host viruses without experiencing severe disease.

## BAT ADAPTATIONS TO TOLERATE VIRUS INFECTION AND MITIGATE INFLAMMATION

Some bat species within the Rhinolophidae and Pteropodidae families host and shed viruses like SARS-CoV-2 and Nipah virus. It has been hypothesized that the long-term coexistence of bats and viruses may have imposed strong selective pressures on the bat genome (22, 23). The genes that are perhaps most likely to reflect this evolutionary selective pressure are directly related to the innate immune system, which is the first line of antiviral defense (24–26). Pattern recognition receptors (PRRs) are critical sensors of the innate immune system and detect pathogen-associated molecular patterns (PAMPs) and damage-associated molecular patterns (DAMPs). Innate immune sensing of viral PAMPs promotes type-I interferon (IFN)-mediated responses. Interferon regulatory factors (IRFs) are transcriptional factors that undergo dimerization and translocate to the nucleus when cellular receptors sense PAMPs, initiating the expression of type I IFNs such as IFNα and IFNβ and consequently expressing interferon-stimulated genes (ISGs) that elicit an antiviral response. Unlike humans, kidney cells from *Pteropus alecto* bats (black flying fox) constitutively express baseline levels of IFNα and ISGs, which may help in controlling virus replication (13, 24). Several innate immune sensors that sense PAMPs regulate inflammation and cytokine response. One important innate immune sensor is the inflammasome complex, which plays an important role in innate immune sensing of various PAMPs and activates the caspase-dependent pro-inflammatory responses (27). Multiple studies have confirmed that bats have dampened inflammasome activation due to dampened NLRP3 (nucleotide-binding and oligomerization domain [NOD], leucine-rich-repeat-containing family [LRR], and pyrin domain-containing protein 3) expression and function (28). This ability to dampen the pro-inflammatory response and subsequent inflammation may be critical for the role of bats as viral reservoirs (13, 14, 28, 29).

The elevated metabolic requirements associated with flight have been anticipated to promote oxidative phosphorylation, leading to the generation of reactive oxygen species (ROS) (30). High levels of ROS can cause severe damage to various cellular components, releasing damaged mitochondrial and nuclear DNA into the cytoplasm (31, 32). The innate immune mechanisms in some bat species have evolved to tolerate these stressors, avoid immune hyperactivation, and mitigate the induction of inflammatory pathways (14, 33, 34).

Unlike other mammals, bats belonging to the families Pteropodidae, Rhinolophidae, Vespertilionidae, Megadermatidae, Mormoopidae, and Molossidae have altered DNA sensing pathways that may limit inflammation caused by damaged DNA present in the cytosol (35, 36). One important cytosolic DNA sensing mechanism involves the stimulator of interferon genes (STING) protein, a vital IFN pathway regulator mutated in *Tadarida brasiliensis* (36). Moreover, genomic analysis of 10 different bat species representing both suborders shows the loss of critical components of the DNA sensing pathway, including the PYHIN (Pyrin and Hin domain) family genes, which initiate inflammatory responses in humans and mice (37). Also, a limited number of mammals and birds are known to show loss of expression of cell death molecules (38–40). Unlike these species, some bat species have evolved alterations in cell death pathways (instead of loss of expression) and show complete loss of all the PYHIN proteins (14, 28, 29, 37) Thus, bats have evolved to alleviate cytosolic DNA-induced innate immune activation and inflammation, enabling virus replication without significant immunopathological symptoms (Fig. 1B). Perhaps, bats might have evolved to regulate robust DNA repair mechanisms to tolerate high levels of ROS generated during flight (20, 30). Understanding these molecular mechanisms behind bats’ viral tolerance is crucial for predicting viral evolution, preparing for future zoonotic spillovers, and developing novel strategies to mitigate the impact of emerging infectious diseases and pandemics.

## CELL DEATH, INFLAMMASOME ACTIVATION, AND VIRAL PATHOGENESIS

Innate immune mechanisms, including regulated cell death (RCD) pathways, act as the body’s first line of defense against pathogenic infections. RCD is a molecularly intricate process essential for destroying viral niches, clearing damaged cells, and mounting an immune response (27). PRRs initiate signaling cascades that activate RCD pathways, including pro-inflammatory pathways such as necroptosis, pyroptosis, and PANoptosis, which differ from the non-inflammatory apoptotic cell death (41, 42). Necroptosis and pyroptosis are lytic cell death modalities characterized by cell swelling and permeabilization of the cell membrane, along with the release of DAMPs and cytokines, which promote inflammation (27). Released DAMPs and cytokines play a significant role in alarming the neighboring cells, mounting an effective immune response by recruiting the immune cells to the site of infection and clearing the virus. However, excessive or uncontrolled cell death and inflammation in humans and mice will promote disease severity in viral infection (43–45).

Specific PRRs sense various PAMPs and DAMPs, activating multimeric complexes known as inflammasomes, ultimately leading to pyroptosis activation and release of pro-inflammatory cytokines. Pyroptosis is mediated by different inflammasomes, such as NLRPs, including NLRP3, the PYHIN domain-containing proteins, such as the absent in melanoma 2 (AIM2), and the pyrin inflammasome (46). Inflammasome multiprotein complexes consist of a PRR molecule (NLRP3, AIM2, etc.), an adaptor protein, and a caspase. PRR is crucial for sensing PAMPs and DAMPs, and the adaptor protein serves as a platform for the complex assembly. The caspase is responsible for the proteolytic cleavage of various downstream molecules promoting pyroptosis and inflammation. The adaptor protein, apoptosis-associated speck-like protein containing a CARD (ASC), includes the N-terminal pyrin domain and the C-terminal caspase activation and recruitment domain (CARD) (45, 47). These domains are essential components of the inflammasome complex. ASC interacts with the NLRP3 and caspase-1 to trigger the activation of caspase-1 via autocatalytic cleavage. Active caspase-1 cleaves downstream molecules such as pro-interleukin-1β (pro-IL-1β) and pro-IL-18, releasing the mature forms. IL-1β and IL-18 are leaderless cytokines facilitating inflammatory responses and are associated with inflammatory RCD pathways, mainly pyroptosis. The absence of a leader sequence ensures their controlled release only via membrane pores or lytic cell death, thereby influencing their functions in immune responses (45).

**Fig 2:**
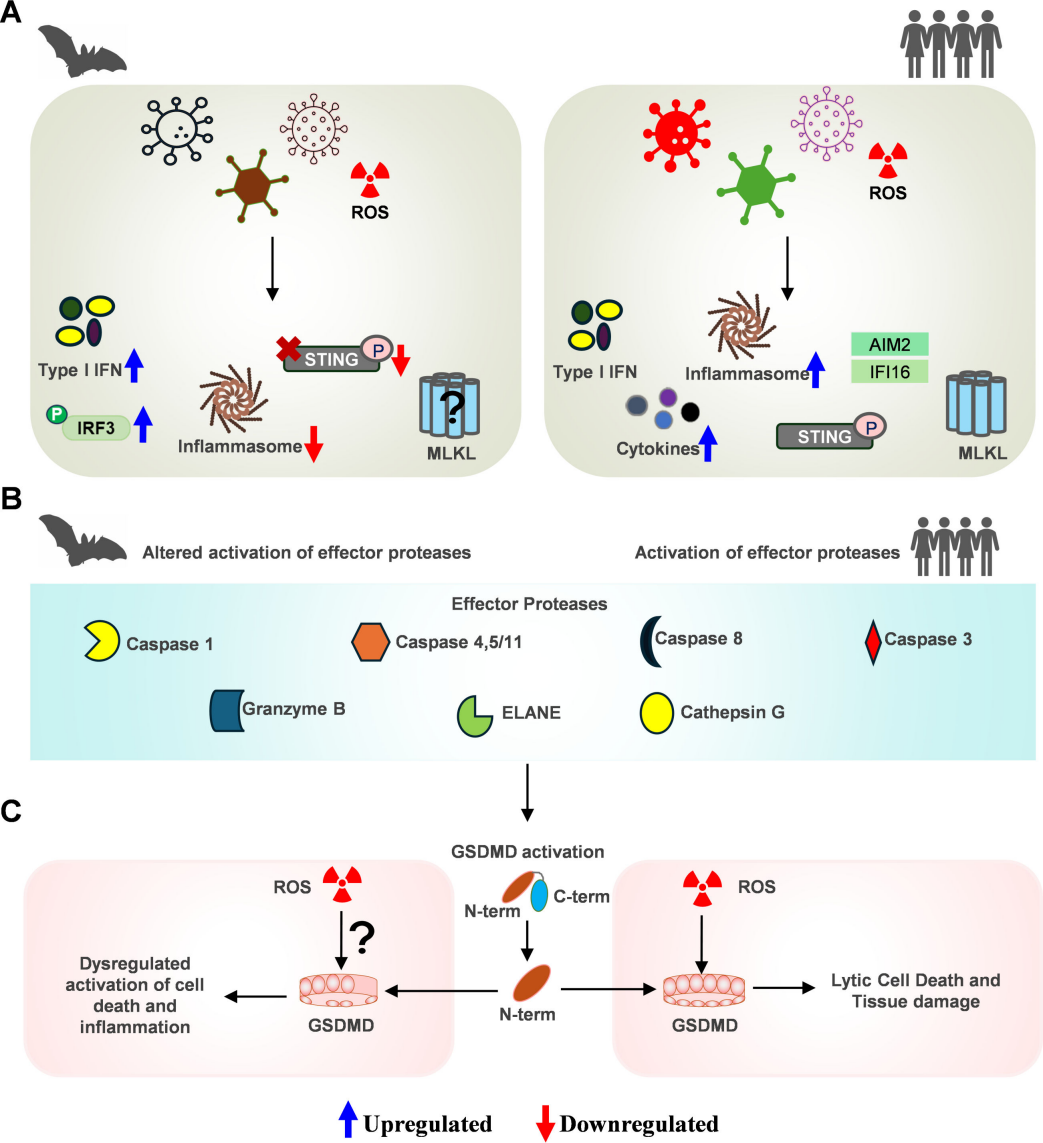
Differential GSDMD response to viral infection and ROS stimuli in humans and bats. (**A**) Humans show a heightened inflammatory response upon any viral infection or ROS stimuli by activating key innate immune and cell death pathways such as necroptosis and pyroptosis. Unlike humans, bats have a dampened inflammatory response due to altered regulation of crucial innate immune and cell death molecules. (**B**) In humans, upstream innate immune pathways activate effector proteases like caspases, granzymes, and cathepsins, which cleave gasdermin family proteins, mainly GSDMD. Upon cleavage, the N-term GSDMD forms a pore on the membrane and promotes pyroptosis. Bats likely show similar GSDMD activation to humans, but this is currently speculation, and mechanistic studies are required to characterize GSDMD in bats. (**C**) ROS directly enhances the activity of GSDMD, leading to increased pyroptosis. In bats, the elevated metabolic requirements associated with flight might promote the generation of reactive oxygen species. Bats possess specific variations in critical residues of GSDMD, plausibly reducing inflammation and limiting tissue damage.

The pore-forming family proteins, gasdermins (GSDMs), are the sole executioners of pyroptosis (48). GSDMs consist of two domains: the N-terminal domain (NTD), responsible for inducing cell death, and the C-terminal domain (CTD), which inhibits gasdermin activation during the resting state. The gasdermin D (GSDMD) is the most studied executioner of pyroptosis, which plays a crucial role in virus infections (49–51). GSDMD is activated by various proteases like caspase-1, 4, and 5 in humans and caspase-1 and 11 in mice (Fig. 2A and B) (52, 53). Caspase-8 also cleaves GSDMD and triggers pyroptosis (53). Upon caspase-mediated cleavage of GSDMD, the NTD assembles into a pore structure on the membrane, facilitating the release of mature forms of IL-1β and IL-18 cytokines and the osmotic lysis (53, 54). The executioner GSDMs can also be activated by inflammasome-independent proteases like elastases, cathepsin G, and granzymes (55–58).

Necroptosis is another inflammatory RCD pathway that is activated when specific receptors, like tumor necrosis factor receptor 1 (TNFR1), toll-like receptors (TLRs), and Z-DNA binding protein 1 (ZBP1), detect specific signals. ZBP1 detects viral Z-nucleic acids and plays a key role in the virus infection and the activation of necroptosis (43, 59–61). The key molecules involved in this process include receptor-interacting protein kinase 1 (RIPK1), RIPK3, and mixed lineage kinase domain-like pseudo kinase (MLKL) (62). RIP-homotypic interaction motifs (RHIMs) are protein motifs that promote homotypic interactions of cell death-associated proteins (63). RHIM-dependent interaction between RIPK1 and RIPK3 or ZBP1 and RIPK3 promotes the assembly of necrosome complex. The activated RIPK3 in the necrosome phosphorylates the effector protein, MLKL, which undergoes oligomerization on the plasma membrane, leading to pore formation and execution of necroptosis, releasing DAMPs and pro-inflammatory cytokines (Fig. 2) (41, 62).

Upon infection, virus-infected cells activate PANoptosis, which recruits inflammatory immune cells to the site of infection and helps in the clearance of viral replication niches (42). PANoptosis is an inflammatory cell death pathway triggered by a multi-protein complex called PANoptosome (41, 42). PANoptosomes primarily comprise sensor proteins, adaptor molecules, and effector proteins. Sensor proteins of PANoptosomes include NLRP3, NLRC5 (NOD-like receptor family CARD domain containing 5), NLRP1, ZBP1, and AIM2, which sense various PAMPs and recruit adaptor molecules such as Fas-associated death domain protein and ASC to create a platform for the activation of effector molecules such as RIPK3 and caspase-8 (42). Controlled activation of PANoptosis effectively clears viral pathogens, but hyper-activation of PANoptosomes can lead to excessive cell death, causing aggravated inflammation and tissue damage in humans and mice (42).

## BAT ADAPTATIONS IN REGULATING INFLAMMASOME AND CELL DEATH PATHWAYS

Over the past decade, a series of comparative genomics and experimental studies on bat immune cells have highlighted the crucial role of adaptations within the bat inflammasome to mitigate pro-inflammatory responses (22, 28, 29, 37). Recent studies have increasingly demonstrated the activation of pyroptosis, which is mediated by inflammasome assembly, particularly the NLRP3 inflammasome, in viral infections in experimental mouse models and human diseases (47, 64). Inflammasome activation has been observed in infections of the influenza A virus, paramyxoviruses, SARS-CoV, MERS-CoV, and, more recently, SARS-CoV-2 (65–68). In *P. alecto*, NLRP3 inflammasome activation is dampened at transcriptional and protein levels (28). Under various TLR agonists, including LPS, Pam3CSK4, and poly(I:C), *P. alecto* bone-marrow-derived dendritic cells, bone-marrow-derived macrophage, and peripheral blood mononuclear cells did not upregulate the transcription of *NLRP3* through the NF-κB pathway, which is a rate-limiting step in NLRP3-mediated inflammasome formation, whereas the basal levels in bat cells were similar to mouse and human primary immune cells (28). Further transcriptomic analysis revealed four isoforms of *P. alecto* NLRP3 with limited activity independent of ASC. Exon 7-negative alternative splicing isoforms of NLRP3 further dampened the inflammasome activity, as validated in two distant bat (*P. alecto* and *Myotis davidii*) kidney cells. In various primary immune cells of *P. alecto*, the dampened release of IL-1β and ASC speck formation after infection with H1N1 influenza A virus, PRV3M virus (a double-stranded RNA [dsRNA] Melaka virus), and MERS-CoV infection further confirmed a dampened inflammasome pathway in these bats (Fig. 1B). Most importantly, the lack of inflammasome activation had minimal effect on the viral load in *P. alecto* immune cells (28).

Analysis of several bat genomes spanning both Yinpterochiroptera and Yangochiroptera suborders revealed the complete loss of the PYHIN gene family in nine bat genomes (22, 37). AIM2 is a member of the PYHIN gene family along with IFI16 (interferon gamma inducible protein 16), and it acts as a sensor for the pathogen-derived DNA (69). Both AIM2 and IFI16 oligomerize and recruit the adapter ASC to form the inflammasome and can activate the downstream pro-inflammatory caspase-1 (70). Deletion of the entire PYHIN gene family across multiple bat species highlights adaptations in the DNA-sensing pathway to reduce inflammasome activation and further downstream inflammation. Intriguingly, AIM2 is absent in several other mammalian species, especially in cattle, dolphins, and platypus, suggesting that the loss of AIM2 might not be a unique adaptation in bats alone (39). Bats have adapted mechanisms to regulate the inflammasome pathway downstream of NLRP3 and AIM2 (29, 71). NLRP3 and AIM2 recruit the adapter ASC and activate caspase-1 (70). Both caspase-1 and IL-1β were dysfunctional to a different extent in *P. alecto* (71). Caspase-1 in *P. alecto* has undergone positive selection where amino acid at the 365th position has changed from Asp to Asn and at the 371st position from Arg to Gln within a specific domain of caspase-1, which is responsible for the homodimerization of caspase-1 and its subsequent activity to cleave IL-1β and GSDMD (72, 73). However, in *M. davidii*, caspase-1 remains fully functional. *IL-1β* was mutated at multiple residues and was the least cleavable from pro-IL-1β to IL-1β by both the human and *M. davidii* caspase-1 (Fig. 1B) (71). *Eonycteris spelaea* had partially dysfunctional caspase-1 and IL-1β, suggesting complementary dampening of the interleukin-converting enzyme (caspase-1) and the interleukins in multiple bat species. Thus, while differences may lie in the evolution of a dampened inflammasome across different bat species, a combination of adaptations within the bat inflammasome drives a weaker inflammatory process in multiple bat species. Furthermore, the selection pressures that drove the differential evolution of the bat inflammasome across different bat species and between bats and other mammals pose an intriguing research question. Birds are the natural reservoirs of influenza viruses (74). A recent study suggests that several avian species show loss of inflammasome sensors and the adapter ASC, except NLRP3 (39). Thus, bats are unique in regulating inflammasome complexes and show dampened NLRP3 inflammasome activation (28). Birds still retain the expression of NLRP3 and might operate NLRP3 inflammasome independent of ASC.

A recent study on bats revealed the presence of a unique ASC2 variant that differs in sequence from its counterparts in other mammals (29). *P. alecto* ASC2 shows enhanced expression and function compared to its human counterpart. Comparative studies showed that bat ASC2 inhibits human inflammasomes via ASC with greater potency than human ASC2, significantly reducing cell death and IL-1β secretion in mouse macrophages. Site-directed mutagenesis of four residues based on human ASC2 in bat ASC2 (E10K, R37E, C61Y, and G77R) disrupted inflammasome inhibition capability, highlighting the role of these four critical residues in a gain of function of bat ASC2 (29). Additionally, *P. alecto* ASC2 decreased sterile and virus-induced inflammation in mouse models. The study also highlighted the role of bat ASC2 and inflammasome in controlling the SARS-CoV-2-induced inflammation in bats (Fig. 1B). While most mammals have lost this gene, bats have maintained it, with high endogenous expression, particularly in myeloid cells like monocytes and macrophages, contrasting with minimal expression in humans.

Genomic analysis of necroptotic proteins in eight different bats (*P. giganteus*, *H. armiger*, *D. rotundus*, *R. sinicus*, *S. hondurensis*, *P. discolor*, *M. natalensis*, and *M. molossus*) from both suborders, suggested necroptosis is similarly regulated to prevent local inflammation (75). Multiple genomics papers and our group recently confirmed the presence of all the proteins involved in necroptosis, mainly RIPK1, RIPK3, ZBP1, and MLKL, in *T. brasiliensis* species (40, 76, 77). A recent study demonstrates a positive selection in primates and bats RIPK3 and MLKL, which is driven by pathogen antagonism, suggesting adaptation against multiple pathogen inhibitors (Fig. 2A) (76). Poxviruses have evolved MLKL homologs (viral MLKL) that bind to RIPK3, blocking necroptosis; however, not all poxviruses exhibit this inhibition and depend on the host specificity (75, 76). With multiple poxviruses being discovered in bats and the ability of poxviruses to undergo rapid horizontal gene transfer events, genes in bats might have evolved viral homologs as a critical host defense factor, subverting cell death (78). Also, we have recently reported that *T. brasiliensis* lung cells (Tb1-Lu) exhibit detectable levels of RHIM proteins such as RIPK1, RIPK3, and ZBP1 (77). Consequently, it has been shown experimentally that Tb1-Lu cells undergo RHIM-mediated necroptosis and apoptosis. Tb1-Lu cells showed a higher apoptotic response to a viral RHIM of SARS-CoV-2, in contrast to the activation of necroptotic cell death in human and mouse cell lines (77). As the RHIM domain is conserved across vertebrates, with one of the core functions of RHIM-containing proteins being the activation of the transcription factor NF-κB, RIP kinases might have evolved to act as a critical mediator to activate the NF-κB pathway rather than activate necroptosis in bats (79). Tb1-Lu cells also exhibit lower cell death levels upon treatment with various apoptotic and necroptotic cell death triggers in contrast to human cells (77). The coevolution of host cell death proteins and viral factors to limit the inflammatory RCD pathways in bats reflects the extensive adaptation strategies to host viruses without showing significant immunopathology.

A persistent MERS-CoV infection model in the bat *Eptesicus fuscus* kidney cells (Efk) repeatedly selected viral variants mutated to sustain higher levels of type I interferons and anti-apoptotic transcripts. In contrast, MERS-CoV infection in human cell lines resulted in cell death (80). Multiple cell death pathways, including the inflammatory pyroptosis, necroptosis, and PANoptosis pathways, trigger the pore-forming protein GSDMD to release DAMPs that exacerbate inflammation. Further research is needed to explore how different arms of inflammatory RCD pathways, particularly the critical executioner GSDMD, are modulated in bats (Fig. 2B) (81, 82). Our research group previously showed that GSDMD in multiple bat species is mutated at critical residues essential for its function (83). The positively charged residues in the α1-helix of the GSDMD N-terminal domain are crucial for membrane interaction and pore assembly (83). In bats, while the caspase-1 cleavage site in GSDMD is conserved, mutations in the GSDMD N-terminal domain may limit pore formation and pyroptosis, potentially altering the immune response (14). Multiple other GSDMs, including GSDMA, GSDMB, and GSDME, have implications in cancer and autoimmune diseases and thus need further studies in bats (48). An intriguing question is why bats may have evolved to dampen inflammatory cell death pathways and whether this is unique among bats. Perhaps controlled activation of inflammatory RCD pathways helps bats mitigate excessive inflammation that could lead to tissue damage, allowing them to tolerate high viral loads without immunopathological signatures. These adaptations will also likely alleviate the potential high metabolic rate-induced cellular damage and inflammation during flight in bats and facilitate the physiological demands of flight without compromising organismal homeostasis.

## BAT ADAPTATIONS IN REGULATING OTHER INFLAMMATORY SIGNALING PATHWAYS TO LIMIT INFLAMMATION

Bats possess unique adaptations in other key inflammatory signaling pathways, allowing them to regulate immune responses and limit excessive inflammation during viral infections. These adaptations potentially influence the cell death and pro-inflammatory responses associated with virus-induced immunopathology. One such adaptation is the variation of the STING pathway, which is necessary for detecting cytosolic DNA and initiating the inflammatory response. In most mammals, STING is activated by cyclic GMP-AMP (cGAMP) in response to cytosolic DNA, triggering the production of type I IFNs (36). However, *T. brasiliensis* possesses a mutation at the S358 residue of the STING protein, which dampens its phosphorylation and ability to activate downstream molecules, thereby reducing the IFN response (35). This adaptation may help bats avoid the excessive detection of cytosolic DNA and DNA-sensing-mediated inflammatory responses (36). In contrast, the increased basal level expression of IFNs and ISGs primes certain bats’ immune systems to respond rapidly to viral infections. The contraction of IFNα and the expansion of IFNω in some species indicate a diverse range of IFN responses tailored to different bat species-specific ecological niches and viral exposures (24). *P. alecto* interferon pathways also exhibit unique regulatory features. The transcription factors IRF7 and IRF3 are crucial for controlling the IFN response. IRF7 shows distinct expression patterns in different tissues, and IRF3 has undergone positive selection at the 185-residue, enabling rapid activation of interferons and conferring antiviral resistance (Fig. 1B) (25, 26). Further research on MERS-CoV highlights the distinct antiviral responses of bat cells. In contrast to human cells, MERS-CoV replication is reduced in bat cells, where infection significantly increases IFNβ transcript levels (84). Interferons are responsible not only for antiviral responses but also for activating cell death pathways (85). Their detection through specific receptors initiates the activation of various cell death-related molecules, including caspases, RIPK1, ZBP1, MLKL, and RIPK3, as well as triggers like inducible nitric oxide synthase (86–88). Since bats show altered regulation of interferons, their downstream cell death pathways have also likely been altered. However, comprehensive studies to verify this are yet to be done.

Genomic studies have identified c-REL, a gene that regulates immune responses and DNA damage repair, as positively selected in bats (89). This gene likely evolved in response to the demands of flight, which imposes oxidative stress and frequent DNA damage. *E. fuscus* kidney cells (Efk) stimulated with TLR ligands showed high levels of IFNβ and low levels of TNFα, in contrast to human cells, where both IFNβ and TNFα are upregulated under similar conditions (89). The suppression of TNFα by c-Rel in bats may limit inflammation and reduce the risk of immunopathology during viral infections, enabling bats to mount an efficient antiviral response while minimizing harmful inflammation (Fig. 1B) (89). Higher expression of the ABCB1 transporter was observed in *P. alecto* cells, which efficiently fluxes out genotoxic compounds, contributing to reduced DNA damage in bats (90). In addition, bats exhibit unique immune features in natural killer cell receptors, antibody binding, and major histocompatibility complex (MHC) molecules (91, 92). The MHC class I in *P. alecto* has a three-amino-acid insertion that enhances peptide binding, aiding in viral control, while MHC class II polymorphism across species indicates strong immune diversity (92, 93). Thus, bat adaptations are not limited to innate immune pathways and show extensive adaptations in adaptive immune pathways.

Bats also rely on autophagy, a cellular process degrading and recycling damaged components, to manage viral infections and maintain cellular homeostasis. Enhanced autophagic responses in *P. alecto* cells infected with viruses like the Australian bat lyssavirus help clear infections and prevent cellular damage, contributing to bats’ status as viral reservoirs (94). These adaptations, which regulate inflammatory signaling pathways, allow bats to coexist with viruses without experiencing significant inflammation, making them an essential subject of study for understanding viral tolerance and zoonotic transmission (94). Heat shock proteins are essential in bat immunity, aiding protein folding under oxidative stress and modulating viral infections by acting as receptors (95). Combined with regulated autophagy and unique temperature-sensitive antibodies, these adaptations underscore bats’ ability to balance immune defense and tolerance, enabling them to host viruses without experiencing severe inflammation or disease.

## TISSUE DAMAGE MITIGATION IN BATS

An essential adaptation that allows bats to sustain persistent viral infections is their ability to mitigate tissue damage and avoid significant inflammation. Recent studies have provided valuable insights into how bats maintain their status as viral reservoirs by examining infections with viruses like coronaviruses, rabies, Marburg, Ebola, Lloviu, and Nipah viruses across different bat species, demonstrating that bats can carry these viruses with minimal immunopathological symptoms (1, 2, 96). In Jamaican fruit bats, *Artibeus jamaicensis*, MERS-CoV replicates efficiently without causing significant disease symptoms (97). Despite the increased virus load, bats exhibited no clinical signs of illness, such as weight loss or fever. This suggests that Jamaican fruit bats have no barriers to MERS-CoV replication at both the receptor and cellular levels. Yet, they can avoid the severe pathology typically associated with the virus in humans.

Further insights into bat-virus interactions have been gained through studies using bat organoid models (three-dimensional cultures) derived from bat tissues that mimic the architecture and function of real organs. Research on *Carollia perspicillata* airway organoids revealed that H1N1 and H3N2 swine influenza viruses could replicate efficiently (98). At the same time, H1N1 showed higher viral loads than H3N2, which caused more significant tissue damage in the organoids. This indicates that while *C. perspicillata* respiratory epithelium may not present a barrier to influenza viruses, the level of tissue damage can vary depending on the viral strain. The ability of H1N1 to replicate without causing as much inflammation as H3N2 suggests a potential adaptation in bats that minimizes the impact of certain viral infections, even when the virus is present in large quantities. These adaptations are not only limited to the respiratory epithelium but extend to bats’ intestinal tissues as well. Studies on *Rhinolophus sinicus* (Chinese horseshoe bats) intestinal organoids demonstrated a heightened antiviral defense mechanism, characterized by a higher basal expression of antiviral genes and a more potent induction of type III IFNs when infected with viruses such as SARS-CoV-2 and CoV-HKU4 and EV-71 (99). Compared to human organoids, bat intestinal organoids respond more rapidly and robustly to viral infections, which may explain why bats experience less tissue damage and inflammation during such infections (99).

Marburg virus (MARV) is another virus that interacts uniquely with bats. Egyptian rousette bats (ERBs), natural hosts for MARV, exhibit limited pro-inflammatory responses upon infection. Although MARV replicates in various tissues of infected ERBs, histopathological analysis reveals only minimal microscopic lesions. For instance, infected ERB samples show discrete inflammatory foci in the liver with some hemorrhages and low numbers of apoptotic or necrotic cells. Still, overall, the tissue damage is limited compared to what is seen in other infected hosts (100). This suggests ERBs have evolved mechanisms to control the spread and impact of MARV, allowing them to survive the infection without the severe consequences observed in other animals and humans. The presence of mononuclear phagocytes and T cells in these inflammatory foci indicates that the immune system is actively managing the infection but in a way that prevents widespread tissue damage (100). The ability of bats to harbor viruses without showing clinical symptoms is also evident in their interactions with rhabdoviruses, including the rabies virus. Various rhabdoviruses have been found in healthy bats, suggesting that these animals often experience asymptomatic infections (101). In particular, European bat lyssavirus 1 (EBLV1) is responsible for more than 95% of cases of human exposure to lyssaviruses in Europe, yet it causes no apparent disease in infected bats (101). The epidemiology and pathogenesis of EBLV1 in the *Eptesicus isabellinus* (meridional serotine bat) highlight the role of bats as reservoirs of viruses that pose a risk to other species, including humans (101).

One of the most striking examples of bats’ extraordinary tolerance to viral infections is seen in their response to the Nipah virus. Nipah is a highly pathogenic virus and can cause severe disease in humans and other animals (8, 10). In a study involving 17 *Pteropus poliocephalus* (grey-headed fruit bats) inoculated with Nipah virus isolated from a human, the bats showed remarkable tolerance compared to other infected animals. Despite infection, bats remained clinically well throughout the study, with no significant febrile responses in contrast to guinea pigs, which exhibited varying degrees of clinical signs, leading to their euthanasia at different times (102). Histological examinations revealed mild lesions in some bats, including chronic interstitial nephritis and focal hepatitis, indicating a subclinical infection. Infected bats exhibit subclinical infections with episodic viral excretion, which may contribute to viral maintenance in wildlife populations.

Research on the rabies virus and Tacaribe virus, in Jamaican fruit bats shows that not all viral infections in bats are asymptomatic (103, 104). Different virus variants can lead to varying infection outcomes (104). For instance, Jamaican fruit bats infected with the N2c rabies variant experienced severe neurological symptoms and widespread neuronal infection, while those infected with the B2c variant showed milder disease and slower viral spread. N2c infects more neurons across different brain areas, whereas B2c mainly affects neuronal cell bodies. The immune response in N2c-infected bats is robust and is associated with inflammatory cell infiltration and neuronal apoptosis (104). Thus, understanding bat-virus interactions is critical to reveal the cellular processes that mitigate tissue damage in these bats. The asymptomatic or clinically mild nature of virus infections in bats may allow the viruses to persist in bat populations with negligible pathological outcomes, thereby maintaining a reservoir for potential spillover events. Understanding these dynamics is crucial for managing viral outbreaks and protecting public health.

## STRATEGIES IN BATS FOR EFFECTIVE ROS HANDLING AND PREVENTING DNA DAMAGE

Bat species, like *Rhinolophus ferrumequinum*, *Myotis myotis*, and *Myotis brandtii*, are considered exceptions to the rate of living theory, which indicates an inverse relationship between body size and metabolic rate (105, 106). Bats are unique mammals having flight function, a metabolically intensive behavior that likely imposes significant oxidative stress on the cells (107, 108). Bats have evolved to adapt specific enablers in their oxidative phosphorylation pathways under positive pressure. Therefore, many of the mitochondrial and nuclear coded OXPHOS genes in bats are under positive selection, indicating that these genes have been optimized to handle high ROS byproducts generated during flight (109). A study on *Myotis lucifugus* (little brown bat) suggested unique mitochondrial efficiency as they produce significantly lower hydrogen peroxide per unit of oxygen consumed than other species (110). This reduced production of free radicals may be a crucial factor contributing to bats’ extended lifespan, aligning with the oxidative stress theory of aging. However, the metabolic cost of bat flight is comparable to that for birds: oxygen consumption during a high-speed flight in several bat and avian species was, on average, four times higher than the preflight rate and more than an order of magnitude above the resting rate (108, 111, 112). Remarkably, the oxidative stress marker level was higher in flying bats and lower at rest, which points to a recovery mechanism found in navigating birds (30). This implies that rest is essential for bats to recover from the oxidative stress of flight and remain fit and long-lived.

Interestingly, while the activity of the antioxidant enzyme superoxide dismutase in bats is comparable to that of other small mammals like shrews and mice, bats exhibit significantly lower levels of protein carbonyls in both soluble and insoluble liver protein fractions (21, 113). This suggests that bats experience less oxidative damage, indicating more efficient protein homeostasis. Further studies have demonstrated that bats experience more oxidative damage from bacterial and fungal infections than viral infections. This is evident from significant differences in reactive oxygen metabolite levels observed 24- and 48-hour post-infusion in bats exposed to infection-mimicking agents like LPS, zymosan, and poly(I:C) (114). Despite their small size and high metabolic rate, long life span of bats might be attributed to their mitochondrial efficiency, reduced oxidative damage, and advanced antioxidant defenses, which provide critical insights into the biological mechanisms of aging.

## CONCLUSIONS

A few decades ago, bat research was relatively nascent, but it has gained significant momentum due to their role as reservoirs for highly pathogenic viruses. Bats possess unique biological traits, including flight and long lifespans, coupled with distinct immune mechanisms that enable them to tolerate high viral loads without suffering from severe disease (Fig. 2A). Ongoing genomic initiatives, such as the Earth BioGenome and Bat1K projects, generate valuable genetic data for comparative studies, while metabolomics, proteomics, and computational models enhance understanding of bat-host interactions. These insights are critical not only for understanding bat immunity but also hold great promise for developing strategies to address viral infections, cancer, aging, and other inflammatory diseases in humans.

Given GSDMD’s role in inflammatory cell death-mediated pathologies, bats may have evolved adaptations in the pyroptotic pathway to mitigate immune response. GSDMD, the executioner of pyroptosis, once thought to be activated only by inflammasomes, can also be activated by multiple other pathways (55, 56). Bats have adapted to dampen inflammasome activation. However, whether bats regulate GSDMD-mediated pyroptosis through inflammasome and other innate immune signaling pathways remains to be established (Fig. 2B). ROS generated during flight activity of bats disrupts cellular homeostasis, triggering mitochondrial dysfunction, lysosomal damage, and ionic imbalances (e.g., potassium efflux) (115). In addition to ROS, these altered intracellular functions can activate the NLRP3 inflammasome. Also, ROS can oxidize and modify components of the NLRP3 complex, promoting its assembly with ASC and pro-caspase-1 (116, 117). ROS has also been reported to directly enhance the activity of GSDMD, leading to increased pyroptosis (Fig. 2C). The GSDMD pore formation is redox-sensitive and is regulated by ROS-dependent palmitoylation in humans and mice, suggesting a possible adaptation of GSDMD in bats (118). During the flight, the high metabolic demands and elevated ROS may enhance GSDMD activity, but mutations in the GSDMD residues of bats could downregulate this process, limiting pyroptosis and inflammation. Additionally, key residues in the GSDMD N-terminal domain required for membrane interaction and pore assembly are mutated in bats to uncharged residues, which may impact pore formation and help bats maintain immune balance (14). More research is needed on GSDMD to fully understand its role in bats’ immune adaptations. Lack of in-depth knowledge of how bat immune adaptations evolved to minimize inflammation and damage from viral infections needs significant attention to understand zoonotic virus biology. Understanding the evolutionary pressures that shaped bat immunity, especially concerning viral evolution and zoonotic spillover, is crucial for future pandemic prevention.
